# Supplementary material for: Analysis of PPARγ Signaling Activity in Psoriasis
Source: Int J Mol Sci. 2021 Aug 10;22(16):8603. doi: 10.3390/ijms22168603 (PMC8395241; doi:10.3390/ijms22168603)
Supplement: Supplementary file 1 [file ijms-22-08603-s001.zip › Supplemental materials_Analysis of PPARg signaling activity in psoriasis/Pathway models/Models images and html files/Differentiation of psoriatic T cells/1012573467.html]

STAT3 ---| GATA3


# Expression STAT3 ---| GATA3

|  |  |
| --- | --- |
| URN | urn:agi-expression:in-out:urn:agi-llid:6774:out:urn:agi-llid:2625:eff=negative |
| Connectivity | 2 |
| References | 2 |
| Effect | negative |

---
